# Editorial: Gone to Pot: Examining the Association Between Cannabis Use and Medical/Psychiatric Disorders

**DOI:** 10.3389/fpsyt.2022.837757

**Published:** 2022-02-08

**Authors:** Rajiv Radhakrishnan, Sinan Guloksuz, Deepak C. D'Souza, Jim van Os

**Affiliations:** ^1^Department of Psychiatry, School of Medicine, Yale University, New Haven, CT, United States; ^2^Department of Psychiatry, Maastricht University Medical Centre, Maastricht, Netherlands; ^3^Department of Psychiatry, University Medical Center Utrecht, Utrecht, Netherlands

**Keywords:** cannabis, psychosis, neurobiology, cannabidiol, microglia

## Introduction

Cannabis continues to be the most widely used drug worldwide, per the 2021 World Drug report. The United Nations Office on Drugs and Crime (UNODC) estimates that ~4% of the global population (i.e., 200 million people) aged 15–64 years used cannabis at least once in 2019. Rates of cannabis use in the past year increased by ~18% over the past 10 years (2010–2019). Additionally the potency of cannabis (with respect to delta-9 tetrahydrocannabinol (THC) concentration) has increased over the past decade. Furthermore, many states and countries have legalized marijuana use for medical purposes and recreational use among adults.

The association between cannabis use and psychosis has been known for over a century (1, 2). Yet our understanding of this association remains incomplete. Multiple lines of evidence ranging from epidemiological studies (3–5) [both cross-sectional and within-subject studies (6)], human laboratory studies using delta-9 tetrahydrocannabinol, the main psychoactive constituent in cannabis (7, 8), and more recently, genetic studies (9, 10) suggest that cannabis use is a risk factor for the later development of psychosis. The association is moderated by additional factors such as onset of use in adolescence, exposure to childhood trauma and other environmental risk factors, and presence of additional symptoms such as perceived stress, anxiety and depression (11–13).

The precise neurobiological underpinnings of this association however remain to be elucidated. In this collection, we bring together some fascinating studies that have attempted to further our understanding of the association between cannabis use and psychosis.

## Papers in this Research Topic

Dawes et al., present a cross-sectional study in 307 individuals examining the association between cannabis use, schizotypy and performance on Kamin blocking. Kamin blocking (KB) is the phenomenon where pre-exposure to a stimulus reduces its ability to be paired with another stimulus in a reinforcement paradigm. KB has been found to be impaired in schizophrenia and to be modulated by dopaminergic activity. Dawes et al. found that neither lifetime nor current cannabis use was associated with the degree of KB. While they found a positive association between total KB score and schizotypy (as measured on Disorganized dimension), the association between degree of aberrant salience and KB was only present in cannabis non-users, but not among cannabis-users. These negative findings in this well-powered study suggest that the Kamin blocking paradigm and aberrant salience are inadequate to explain the association between cannabis use and psychosis.

What can animal models of psychosis tell us about the association between cannabis use and psychiatric disorders? Jenkins and Khokhar, present a concise review of the literature and note that both preclinical models and human studies point to electrophysiological alterations in gamma oscillations with cannabinoids and in psychiatric disorders; and the role of the interaction between cannabinoids and GABAergic interneurons. This insight is consistent with the finding that pharmacologically induced GABAergic deficit, enhanced the psychotomimetic effects of delta-9 THC (14).

The interactive effects of THC and CBD is also an important consideration in deepening our understanding of the neurobiological effects of cannabis. Woelfl et al., present an interesting laboratory study in humans in which they examine the interactive effects of THC and CBD on cognition. They show that THC (20 mg) reduced the cognitive processing speed and impaired the performance on attention, but pre-treatment with CBD (800 mg) did not attenuate these effects.

The effect of cannabis on adolescence, a critical period of neurodevelopment is an important area of research. In an interesting study, Chen and Mackie, treated adolescent (28 days old) C57BL6/J mice of both sexes for 3 weeks with 3 mg/kg tetrahydrocannabinol (THC). Mice treated with THC as adolescents showed impairment in working memory but did not impact social preference, anxiety behaviors, or decision-making behaviors.

Blest-Hopley et al., provide an up-to-date review of the effects of cannabis on adolescence in human neuroimaging studies. The literature notes that adolescence-onset cannabis use is associated with poorer cognitive performance. However, whether these deficits persist following prolonged abstinence is unclear. Functional MRI (fMRI) studies note increased activation and altered brain connectivity. Structural MRI studies however have not been consistent in showing reduction in gray-matter volume. It remains to be seen whether positron emission tomography (PET) is more sensitive in detecting gray-matter changes during adolescence. A recent study using [11C]UCB-J PET, sensitive and specific to synaptic vesicle protein, found that cannabis users had 10% lower hippocampal synaptic vesicle density compared to healthy controls, although there were no significant differences on volumetric measures using structural MRI (15).

The effects of cannabis on brain glial cells is another exciting area that deserves attention since these cells express cannabinoid CB2 receptors. Watts et al., using proton magnetic resonance spectroscopy (1H-MRS), examined neurometabolite alterations in 26 cannabis users. Lower myo-inositol, a putative marker of glial function, was related to greater severity of cannabis use. They also note interesting sex differences, whereby greater past-year cannabis exposure was related to higher concentrations of glutamate metabolites in male cannabis users but not in female cannabis users.

While the vast majority of studies to understand the neurobiological effects of cannabis have focused on the major cannabinoids i.e., delta-9 THC and cannabidiol (CBD), cannabis contains more than 400 different constituents including terpenes and flavonoids. Weston-Green et al., present an extensive review the literature on the effects of terpenes such as pinene and linalool on neurotransmitters and highlight their potential anti-inflammatory, antioxidant and neuroprotective properties.

## Challenges and Future Directions

As is evident from this collection of articles, understanding the association between cannabis use and psychosis is a challenging problem. While epidemiological studies continue to elucidate the complexity of this association with the goal of early identification of high-risk groups, the neurobiological underpinnings of this association is far more elusive. Complicating this problem is the fact that cannabis contains over 400 different chemical constituents, many of which interact directly with the endocannabinoid system, the potency of cannabis has changed with time and psychosis is a heterogenous group of disorders with different neurobiological changes at different stages of the disorder. A clearer understanding of the neurobiological basis of this relationship may hence warrant longitudinal studies that deeply phenotype individuals including genetic risk, environmental exposure and multimodal imaging.

We hope this interesting collection of articles on cannabis and psychosis, piques the readers interest and open up new avenues for research in the coming years.

## Author Contributions

All authors listed have made a substantial, direct, and intellectual contribution to the work and approved it for publication.

## Conflict of Interest

The authors declare that the research was conducted in the absence of any commercial or financial relationships that could be construed as a potential conflict of interest.

## Publisher's Note

All claims expressed in this article are solely those of the authors and do not necessarily represent those of their affiliated organizations, or those of the publisher, the editors and the reviewers. Any product that may be evaluated in this article, or claim that may be made by its manufacturer, is not guaranteed or endorsed by the publisher.
